# *N*-(2-Fluoro­benz­yloxy)-1,3,5-trimethyl-2,6-diphenyl­piperidin-4-imine

**DOI:** 10.1107/S1600536812029327

**Published:** 2012-07-04

**Authors:** Chennan Ramalingan, Seik Weng Ng, Edward R. T. Tiekink

**Affiliations:** a Centre for Nanotechnology, Department of Chemistry, Kalasalingam University, Krishnankoil 626 126, Tamilnadu, India; b Department of Chemistry, University of Malaya, 50603 Kuala Lumpur, Malaysia; c Chemistry Department and Faculty of Science, King Abdulaziz University, PO Box 80203 Jeddah, Saudi Arabia

## Abstract

In the title compound, C_27_H_29_FN_2_O, the piperidine ring has a twisted boat conformation and all ring substituents occupy equatorial positions. The dihedral angle formed between the phenyl rings is 66.71 (12)°, and the phenyl rings form dihedral angles of 46.60 (13) and 43.75 (13)° with the fluoro­benzene ring, which occupies a position coplanar to the meth­oxy(methyl­idene)amine residue [N—O—C—C torsion angle = −179.5 (2)°]. In the crystal, a complex network of C—H⋯π inter­actions connects the mol­ecules into a three-dimensional architecture.

## Related literature

For the biological activity of mol­ecules having a 2,6-diaryl­piperidine core, see: Ramachandran *et al.* (2011 ▶); Ramalingan *et al.* (2004 ▶). For the structures of related chloro and bromo derivatives, see: Ramalingan *et al.* (2012*a* ▶,*b* ▶). For the synthesis, see: Ramalingan *et al.* (2006 ▶).
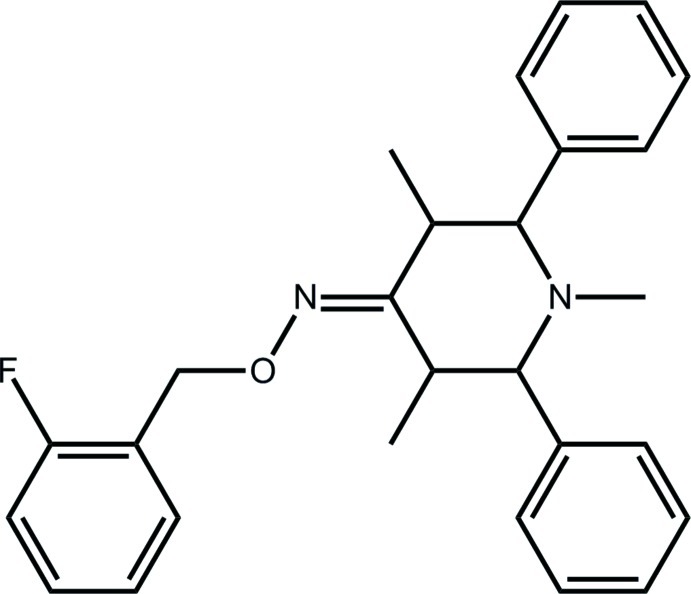


## Experimental

### 

#### Crystal data

C_27_H_29_FN_2_O
*M* *_r_* = 416.52
Orthorhombic, 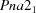

*a* = 7.4004 (3) Å
*b* = 22.4857 (9) Å
*c* = 13.4465 (5) Å
*V* = 2237.54 (15) Å^3^
*Z* = 4
Mo *K*α radiation
μ = 0.08 mm^−1^
*T* = 100 K
0.30 × 0.20 × 0.15 mm

#### Data collection

Agilent SuperNova Dual diffractometer with an Atlas detector
Absorption correction: multi-scan (*CrysAlis PRO*; Agilent, 2012 ▶) *T*_min_ = 0.930, *T*_max_ = 1.000
14812 measured reflections
2693 independent reflections
2311 reflections with *I* > 2σ(*I*)
*R*_int_ = 0.058

#### Refinement

*R*[*F* ^2^ > 2σ(*F* ^2^)] = 0.041
*wR*(*F* ^2^) = 0.101
*S* = 1.03
2693 reflections
280 parameters
1 restraint
H-atom parameters constrained
Δρ_max_ = 0.21 e Å^−3^
Δρ_min_ = −0.24 e Å^−3^

### 

Data collection: *CrysAlis PRO* (Agilent, 2012 ▶); cell refinement: *CrysAlis PRO*; data reduction: *CrysAlis PRO*; program(s) used to solve structure: *SHELXS97* (Sheldrick, 2008 ▶); program(s) used to refine structure: *SHELXL97* (Sheldrick, 2008 ▶); molecular graphics: *ORTEP-3 for Windows* (Farrugia, 1997 ▶) and *DIAMOND* (Brandenburg, 2006 ▶); software used to prepare material for publication: *publCIF* (Westrip, 2010 ▶).

## Supplementary Material

Crystal structure: contains datablock(s) global, I. DOI: 10.1107/S1600536812029327/pv2563sup1.cif

Structure factors: contains datablock(s) I. DOI: 10.1107/S1600536812029327/pv2563Isup2.hkl

Supplementary material file. DOI: 10.1107/S1600536812029327/pv2563Isup3.cml

Additional supplementary materials:  crystallographic information; 3D view; checkCIF report

## Figures and Tables

**Table 1:** Hydrogen-bond geometry (Å, °) *Cg*1–*Cg*3 are the centroids of the C1–C6, C16–C21 and C22–C27 rings, respectively.

*D*—H⋯*A*	*D*—H	H⋯*A*	*D*⋯*A*	*D*—H⋯*A*
C7—H7*A*⋯*Cg*1^i^	0.99	2.96	3.721 (3)	135
C13—H13*A*⋯*Cg*2^ii^	0.98	2.91	3.577 (3)	127
C18—H18⋯*Cg*3^iii^	0.95	2.90	3.700 (3)	143
C21—H21⋯*Cg*2^iv^	0.95	2.51	3.446 (3)	167
C25—H25⋯*Cg*3^v^	0.95	2.74	3.654 (3)	160

Symmetry codes: (i) 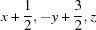; (ii) 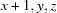; (iii) 
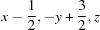
; (iv) 
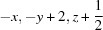
; (v) 
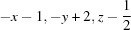
.

**Fig. 1.**
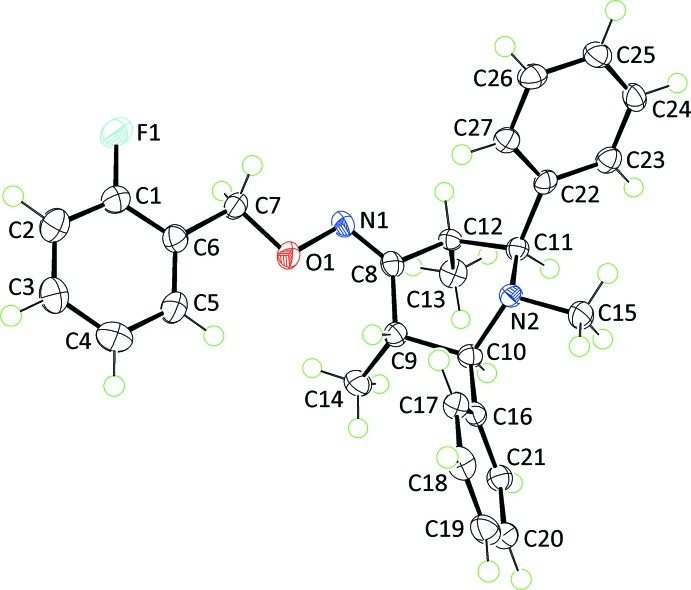
The molecular structure of the title compound showing the atom-labelling scheme and displacement ellipsoids at the 70% probability level.

**Fig. 2.**
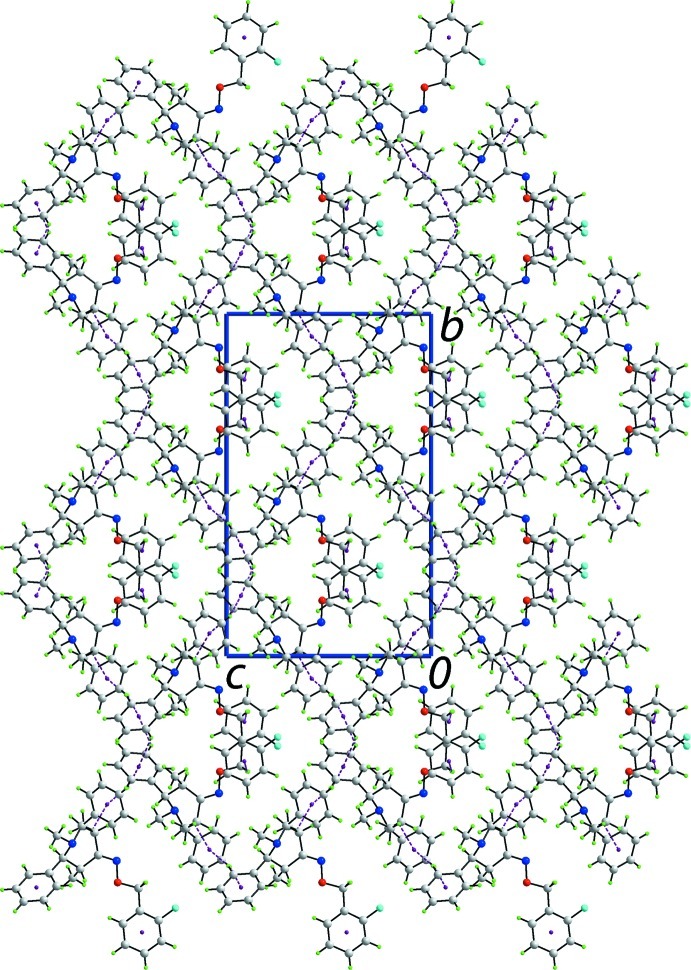
A view in projection down the a axis of the unit-cell contents for the title compound, the C—H···π interactions are shown as purple dashed lines.

## supplementary crystallographic information

### Comment

In a program aimed towards synthesizing efficient biological agents, the title compound, (I), was generated (Ramalingan *et al.*, 2006) as molecules with a 2,6-diarylpiperidine core are known to exhibit potent biological activities (Ramachandran *et al.*, 2011; Ramalingan *et al.*, 2004). Herein, the crystal and molecular structure of the title compound is described.

In the title molecule (Fig. 1), the piperidine ring adopts a twist-boat conformation and all ring-substituents occupy equatorial positions. In the chloro (Ramalingan *et al.*, 2012*b*) and bromo (Ramalingan *et al.*, 2012*b*) analogues of the title compound, which lack a C-bound methyl substituent and where the piperidine ring adopts a chair conformation, all C-bound substituents are found in equatorial positions and the *N*-bound methyl group is in a bisectional position (Ramalingan *et al.*, 2012*a*, 2012*b*). The dihedral angle formed between the C15–C20 and C21–C26 benzene rings in the title compound is 66.71 (12)°, and each forms a dihedral angle of 46.60 (13) and 43.75 (13)°, respectively, with the fluorobenzene ring, which occupies a position co-planar to the methoxy(methylidene)amine residue, as seen in the N1—O1—C7—C6 torsion angle of -179.5 (2)°. In the aforementioned chloro and bromo derivatives, orthogonal and co-planar orientations were observed in this region of the respective molecule, respectively. The conformation about the imine C8═N1 bond [1.278 (3) Å] is *E*.

A complex network of C—H···π interactions connects the molecules into a three-dimensional architecture (Table 1 and Fig. 2).

### Experimental

For full details of the synthesis, refer to Ramalingan *et al.* (2006). Re-crystallization was performed by slow evaporation of an ethanolic solution of (I) which afforded colourless crystals. *M*.pt: 378–379 K.

### Refinement

Carbon-bound H-atoms were placed in calculated positions [C—H = 0.95–1.00 Å, *U*_iso_(H) = 1.2–1.5*U*_eq_(C)] and were included in the refinement in the riding model approximation. In the absence of significant anomalous scattering effects, 2345 Friedel pairs were averaged in the final refinement.

### Crystal data

**Table:**

C_27_H_29_FN_2_O	*F*(000) = 888
*M**_r_* = 416.52	*D*_x_ = 1.236 Mg m^−^^3^
Orthorhombic, *P**n**a*2_1_	Mo *K*α radiation, λ = 0.71073 Å
Hall symbol: P 2c -2n	Cell parameters from 4254 reflections
*a* = 7.4004 (3) Å	θ = 2.4–27.5°
*b* = 22.4857 (9) Å	µ = 0.08 mm^−^^1^
*c* = 13.4465 (5) Å	*T* = 100 K
*V* = 2237.54 (15) Å^3^	Prism, colourless
*Z* = 4	0.30 × 0.20 × 0.15 mm

### Data collection

**Table:**

Agilent SuperNova Dual diffractometer with an Atlas detector	2693 independent reflections
Radiation source: SuperNova (Mo) X-ray Source	2311 reflections with *I* > 2σ(*I*)
Mirror monochromator	*R*_int_ = 0.058
Detector resolution: 10.4041 pixels mm^-1^	θ_max_ = 27.6°, θ_min_ = 2.9°
ω scan	*h* = −9→9
Absorption correction: multi-scan (*CrysAlis PRO*; Agilent, 2012)	*k* = −21→29
*T*_min_ = 0.930, *T*_max_ = 1.000	*l* = −17→16
14812 measured reflections	

### Refinement

**Table:**

Refinement on *F*^2^	Primary atom site location: structure-invariant direct methods
Least-squares matrix: full	Secondary atom site location: difference Fourier map
*R*[*F*^2^ > 2σ(*F*^2^)] = 0.041	Hydrogen site location: inferred from neighbouring sites
*wR*(*F*^2^) = 0.101	H-atom parameters constrained
*S* = 1.03	*w* = 1/[σ^2^(*F*_o_^2^) + (0.0469*P*)^2^ + 0.573*P*] where *P* = (*F*_o_^2^ + 2*F*_c_^2^)/3
2693 reflections	(Δ/σ)_max_ = 0.001
280 parameters	Δρ_max_ = 0.21 e Å^−^^3^
1 restraint	Δρ_min_ = −0.24 e Å^−^^3^

### Special details

**Table:**

Geometry. All e.s.d.'s (except the e.s.d. in the dihedral angle between two l.s. planes) are estimated using the full covariance matrix. The cell e.s.d.'s are taken into account individually in the estimation of e.s.d.'s in distances, angles and torsion angles; correlations between e.s.d.'s in cell parameters are only used when they are defined by crystal symmetry. An approximate (isotropic) treatment of cell e.s.d.'s is used for estimating e.s.d.'s involving l.s. planes.
Refinement. Refinement of *F*^2^ against ALL reflections. The weighted *R*-factor *wR* and goodness of fit *S* are based on *F*^2^, conventional *R*-factors *R* are based on *F*, with *F* set to zero for negative *F*^2^. The threshold expression of *F*^2^ > σ(*F*^2^) is used only for calculating *R*-factors(gt) *etc*. and is not relevant to the choice of reflections for refinement. *R*-factors based on *F*^2^ are statistically about twice as large as those based on *F*, and *R*- factors based on ALL data will be even larger.

### Fractional atomic coordinates and isotropic or equivalent isotropic displacement parameters (Å^2^)

**Table:**

	*x*	*y*	*z*	*U*_iso_*/*U*_eq_	
F1	0.1682 (3)	0.76313 (8)	−0.25008 (12)	0.0369 (5)	
O1	0.1493 (2)	0.83790 (8)	0.03234 (14)	0.0210 (4)	
N1	0.1480 (3)	0.90124 (9)	0.03729 (17)	0.0177 (5)	
N2	−0.1331 (3)	0.95190 (9)	0.25393 (16)	0.0142 (4)	
C1	0.1633 (4)	0.72749 (12)	−0.1681 (2)	0.0222 (6)	
C2	0.1639 (4)	0.66675 (13)	−0.1813 (2)	0.0282 (6)	
H2	0.1681	0.6499	−0.2461	0.034*	
C3	0.1585 (4)	0.63103 (13)	−0.0983 (2)	0.0295 (7)	
H3	0.1583	0.5890	−0.1054	0.035*	
C4	0.1533 (4)	0.65620 (14)	−0.0041 (2)	0.0329 (7)	
H4	0.1498	0.6315	0.0531	0.040*	
C5	0.1534 (4)	0.71756 (12)	0.0062 (2)	0.0259 (6)	
H5	0.1503	0.7345	0.0709	0.031*	
C6	0.1578 (4)	0.75462 (12)	−0.0761 (2)	0.0191 (5)	
C7	0.1582 (4)	0.82144 (11)	−0.06990 (19)	0.0209 (6)	
H7A	0.2699	0.8375	−0.1003	0.025*	
H7B	0.0531	0.8378	−0.1063	0.025*	
C8	0.1264 (3)	0.91809 (11)	0.12724 (19)	0.0154 (5)	
C9	0.1013 (3)	0.87766 (11)	0.21545 (18)	0.0156 (5)	
H9	0.0246	0.8435	0.1933	0.019*	
C10	−0.0016 (3)	0.90997 (10)	0.29954 (19)	0.0153 (5)	
H10	0.0868	0.9332	0.3402	0.018*	
C11	−0.0404 (3)	1.00355 (11)	0.20744 (18)	0.0147 (5)	
H11	0.0010	1.0308	0.2616	0.018*	
C12	0.1286 (3)	0.98411 (11)	0.14558 (18)	0.0149 (5)	
H12	0.1226	1.0046	0.0796	0.018*	
C13	0.3042 (3)	1.00342 (12)	0.1971 (2)	0.0207 (6)	
H13A	0.4080	0.9908	0.1571	0.031*	
H13B	0.3112	0.9850	0.2631	0.031*	
H13C	0.3055	1.0468	0.2042	0.031*	
C14	0.2820 (4)	0.85183 (12)	0.2516 (2)	0.0227 (6)	
H14A	0.3420	0.8313	0.1965	0.034*	
H14B	0.2596	0.8236	0.3058	0.034*	
H14C	0.3594	0.8841	0.2756	0.034*	
C15	−0.2592 (4)	0.97521 (12)	0.3285 (2)	0.0212 (5)	
H15A	−0.3437	1.0028	0.2966	0.032*	
H15B	−0.1915	0.9962	0.3804	0.032*	
H15C	−0.3267	0.9422	0.3583	0.032*	
C16	−0.0951 (3)	0.86507 (11)	0.36609 (19)	0.0146 (5)	
C17	−0.2307 (3)	0.82735 (11)	0.3303 (2)	0.0173 (5)	
H17	−0.2665	0.8297	0.2626	0.021*	
C18	−0.3132 (4)	0.78652 (11)	0.3928 (2)	0.0200 (6)	
H18	−0.4060	0.7614	0.3678	0.024*	
C19	−0.2608 (4)	0.78218 (12)	0.4915 (2)	0.0222 (6)	
H19	−0.3162	0.7537	0.5338	0.027*	
C20	−0.1280 (3)	0.81934 (11)	0.5284 (2)	0.0202 (6)	
H20	−0.0924	0.8167	0.5961	0.024*	
C21	−0.0465 (3)	0.86073 (11)	0.46574 (19)	0.0169 (5)	
H21	0.0439	0.8865	0.4915	0.020*	
C22	−0.1760 (3)	1.03673 (11)	0.14343 (18)	0.0149 (5)	
C23	−0.2317 (4)	1.09396 (11)	0.16686 (19)	0.0178 (5)	
H23	−0.1837	1.1133	0.2239	0.021*	
C24	−0.3578 (4)	1.12344 (12)	0.1073 (2)	0.0197 (6)	
H24	−0.3947	1.1627	0.1239	0.024*	
C25	−0.4290 (4)	1.09576 (11)	0.0244 (2)	0.0210 (6)	
H25	−0.5152	1.1157	−0.0160	0.025*	
C26	−0.3732 (3)	1.03838 (12)	0.0005 (2)	0.0193 (5)	
H26	−0.4219	1.0191	−0.0564	0.023*	
C27	−0.2472 (3)	1.00917 (11)	0.05908 (18)	0.0163 (5)	
H27	−0.2090	0.9702	0.0417	0.020*	

### Atomic displacement parameters (Å^2^)

**Table:**

	*U*^11^	*U*^22^	*U*^33^	*U*^12^	*U*^13^	*U*^23^
F1	0.0700 (14)	0.0239 (9)	0.0167 (8)	0.0023 (9)	0.0004 (8)	−0.0001 (7)
O1	0.0322 (10)	0.0142 (9)	0.0167 (9)	0.0021 (7)	0.0041 (8)	−0.0017 (8)
N1	0.0206 (11)	0.0131 (10)	0.0193 (11)	0.0022 (8)	0.0011 (9)	−0.0004 (9)
N2	0.0139 (10)	0.0147 (10)	0.0140 (10)	0.0006 (8)	0.0016 (8)	0.0014 (8)
C1	0.0282 (14)	0.0208 (14)	0.0178 (13)	0.0010 (11)	0.0016 (12)	0.0011 (11)
C2	0.0357 (16)	0.0258 (15)	0.0232 (15)	0.0015 (12)	−0.0042 (13)	−0.0069 (12)
C3	0.0397 (18)	0.0186 (14)	0.0301 (16)	0.0014 (12)	−0.0004 (14)	−0.0046 (12)
C4	0.0461 (18)	0.0248 (16)	0.0279 (16)	0.0032 (14)	0.0007 (14)	0.0069 (13)
C5	0.0372 (17)	0.0217 (14)	0.0189 (14)	0.0021 (12)	0.0022 (12)	−0.0044 (11)
C6	0.0182 (13)	0.0200 (13)	0.0192 (12)	0.0039 (10)	0.0001 (10)	−0.0017 (11)
C7	0.0320 (15)	0.0170 (13)	0.0137 (12)	0.0016 (11)	0.0044 (11)	−0.0012 (11)
C8	0.0104 (11)	0.0172 (13)	0.0186 (12)	0.0018 (9)	−0.0002 (10)	−0.0008 (10)
C9	0.0159 (12)	0.0149 (12)	0.0160 (12)	0.0006 (9)	0.0029 (10)	0.0008 (10)
C10	0.0146 (12)	0.0159 (12)	0.0152 (11)	0.0000 (9)	−0.0006 (9)	0.0005 (10)
C11	0.0169 (12)	0.0136 (12)	0.0136 (11)	−0.0004 (9)	−0.0008 (10)	−0.0014 (10)
C12	0.0166 (12)	0.0135 (12)	0.0146 (12)	0.0008 (10)	0.0004 (10)	0.0026 (10)
C13	0.0172 (12)	0.0217 (14)	0.0233 (14)	−0.0043 (10)	0.0018 (12)	−0.0005 (12)
C14	0.0210 (13)	0.0249 (14)	0.0220 (13)	0.0073 (11)	0.0010 (11)	0.0037 (12)
C15	0.0219 (13)	0.0211 (13)	0.0206 (13)	0.0044 (11)	0.0074 (11)	0.0033 (11)
C16	0.0146 (12)	0.0132 (12)	0.0161 (12)	0.0022 (9)	0.0025 (10)	0.0009 (10)
C17	0.0186 (12)	0.0172 (12)	0.0161 (11)	0.0014 (10)	−0.0001 (10)	−0.0010 (10)
C18	0.0153 (12)	0.0168 (13)	0.0278 (14)	−0.0022 (10)	0.0014 (11)	−0.0007 (11)
C19	0.0219 (14)	0.0174 (13)	0.0271 (15)	0.0019 (11)	0.0072 (12)	0.0063 (11)
C20	0.0220 (13)	0.0225 (13)	0.0162 (12)	0.0050 (10)	0.0017 (11)	0.0014 (11)
C21	0.0163 (12)	0.0170 (13)	0.0173 (12)	0.0003 (10)	−0.0001 (10)	−0.0001 (10)
C22	0.0160 (12)	0.0153 (12)	0.0135 (12)	−0.0015 (9)	0.0050 (10)	0.0017 (10)
C23	0.0206 (13)	0.0168 (13)	0.0160 (12)	−0.0027 (10)	0.0029 (10)	0.0004 (10)
C24	0.0234 (14)	0.0135 (13)	0.0223 (14)	0.0037 (10)	0.0064 (11)	0.0029 (11)
C25	0.0180 (13)	0.0249 (14)	0.0200 (13)	0.0048 (11)	0.0025 (11)	0.0056 (11)
C26	0.0189 (13)	0.0228 (13)	0.0162 (12)	0.0001 (10)	−0.0035 (11)	0.0006 (11)
C27	0.0177 (12)	0.0148 (12)	0.0164 (12)	−0.0020 (10)	0.0013 (10)	0.0011 (10)

### Geometric parameters (Å, º)

**Table:**

F1—C1	1.363 (3)	C13—H13A	0.9800
O1—C7	1.425 (3)	C13—H13B	0.9800
O1—N1	1.426 (3)	C13—H13C	0.9800
N1—C8	1.278 (3)	C14—H14A	0.9800
N2—C15	1.467 (3)	C14—H14B	0.9800
N2—C11	1.487 (3)	C14—H14C	0.9800
N2—C10	1.487 (3)	C15—H15A	0.9800
C1—C2	1.377 (4)	C15—H15B	0.9800
C1—C6	1.381 (4)	C15—H15C	0.9800
C2—C3	1.376 (4)	C16—C21	1.391 (3)
C2—H2	0.9500	C16—C17	1.399 (4)
C3—C4	1.388 (4)	C17—C18	1.386 (4)
C3—H3	0.9500	C17—H17	0.9500
C4—C5	1.387 (4)	C18—C19	1.387 (4)
C4—H4	0.9500	C18—H18	0.9500
C5—C6	1.386 (4)	C19—C20	1.382 (4)
C5—H5	0.9500	C19—H19	0.9500
C6—C7	1.505 (4)	C20—C21	1.392 (4)
C7—H7A	0.9900	C20—H20	0.9500
C7—H7B	0.9900	C21—H21	0.9500
C8—C9	1.506 (4)	C22—C23	1.388 (3)
C8—C12	1.505 (3)	C22—C27	1.396 (4)
C9—C14	1.536 (4)	C23—C24	1.397 (4)
C9—C10	1.545 (3)	C23—H23	0.9500
C9—H9	1.0000	C24—C25	1.381 (4)
C10—C16	1.516 (3)	C24—H24	0.9500
C10—H10	1.0000	C25—C26	1.392 (4)
C11—C22	1.518 (3)	C25—H25	0.9500
C11—C12	1.564 (3)	C26—C27	1.386 (4)
C11—H11	1.0000	C26—H26	0.9500
C12—C13	1.536 (3)	C27—H27	0.9500
C12—H12	1.0000		
			
C7—O1—N1	107.75 (18)	C11—C12—H12	107.9
C8—N1—O1	110.0 (2)	C12—C13—H13A	109.5
C15—N2—C11	107.57 (18)	C12—C13—H13B	109.5
C15—N2—C10	111.1 (2)	H13A—C13—H13B	109.5
C11—N2—C10	111.54 (18)	C12—C13—H13C	109.5
F1—C1—C2	118.6 (2)	H13A—C13—H13C	109.5
F1—C1—C6	117.8 (2)	H13B—C13—H13C	109.5
C2—C1—C6	123.6 (3)	C9—C14—H14A	109.5
C3—C2—C1	118.3 (3)	C9—C14—H14B	109.5
C3—C2—H2	120.9	H14A—C14—H14B	109.5
C1—C2—H2	120.9	C9—C14—H14C	109.5
C2—C3—C4	120.2 (3)	H14A—C14—H14C	109.5
C2—C3—H3	119.9	H14B—C14—H14C	109.5
C4—C3—H3	119.9	N2—C15—H15A	109.5
C5—C4—C3	119.8 (3)	N2—C15—H15B	109.5
C5—C4—H4	120.1	H15A—C15—H15B	109.5
C3—C4—H4	120.1	N2—C15—H15C	109.5
C6—C5—C4	121.2 (3)	H15A—C15—H15C	109.5
C6—C5—H5	119.4	H15B—C15—H15C	109.5
C4—C5—H5	119.4	C21—C16—C17	118.3 (2)
C1—C6—C5	116.8 (2)	C21—C16—C10	119.8 (2)
C1—C6—C7	119.4 (2)	C17—C16—C10	121.9 (2)
C5—C6—C7	123.8 (2)	C18—C17—C16	120.6 (2)
O1—C7—C6	108.2 (2)	C18—C17—H17	119.7
O1—C7—H7A	110.1	C16—C17—H17	119.7
C6—C7—H7A	110.1	C19—C18—C17	120.2 (3)
O1—C7—H7B	110.1	C19—C18—H18	119.9
C6—C7—H7B	110.1	C17—C18—H18	119.9
H7A—C7—H7B	108.4	C20—C19—C18	120.0 (2)
N1—C8—C9	125.6 (2)	C20—C19—H19	120.0
N1—C8—C12	116.5 (2)	C18—C19—H19	120.0
C9—C8—C12	117.9 (2)	C19—C20—C21	119.7 (3)
C8—C9—C14	111.7 (2)	C19—C20—H20	120.2
C8—C9—C10	110.7 (2)	C21—C20—H20	120.2
C14—C9—C10	112.0 (2)	C16—C21—C20	121.2 (2)
C8—C9—H9	107.4	C16—C21—H21	119.4
C14—C9—H9	107.4	C20—C21—H21	119.4
C10—C9—H9	107.4	C23—C22—C27	119.0 (2)
N2—C10—C16	111.5 (2)	C23—C22—C11	121.6 (2)
N2—C10—C9	108.59 (19)	C27—C22—C11	119.5 (2)
C16—C10—C9	110.12 (19)	C22—C23—C24	120.6 (2)
N2—C10—H10	108.8	C22—C23—H23	119.7
C16—C10—H10	108.8	C24—C23—H23	119.7
C9—C10—H10	108.8	C25—C24—C23	120.2 (2)
N2—C11—C22	108.50 (19)	C25—C24—H24	119.9
N2—C11—C12	111.97 (18)	C23—C24—H24	119.9
C22—C11—C12	111.37 (19)	C24—C25—C26	119.4 (2)
N2—C11—H11	108.3	C24—C25—H25	120.3
C22—C11—H11	108.3	C26—C25—H25	120.3
C12—C11—H11	108.3	C27—C26—C25	120.5 (2)
C8—C12—C13	111.2 (2)	C27—C26—H26	119.7
C8—C12—C11	110.75 (19)	C25—C26—H26	119.7
C13—C12—C11	110.9 (2)	C26—C27—C22	120.4 (2)
C8—C12—H12	107.9	C26—C27—H27	119.8
C13—C12—H12	107.9	C22—C27—H27	119.8
			
C7—O1—N1—C8	175.2 (2)	N1—C8—C12—C13	−107.0 (3)
F1—C1—C2—C3	−179.8 (3)	C9—C8—C12—C13	72.4 (3)
C6—C1—C2—C3	0.2 (4)	N1—C8—C12—C11	129.2 (2)
C1—C2—C3—C4	−0.3 (4)	C9—C8—C12—C11	−51.5 (3)
C2—C3—C4—C5	0.1 (5)	N2—C11—C12—C8	14.6 (3)
C3—C4—C5—C6	0.2 (5)	C22—C11—C12—C8	−107.1 (2)
F1—C1—C6—C5	−179.8 (3)	N2—C11—C12—C13	−109.4 (2)
C2—C1—C6—C5	0.2 (4)	C22—C11—C12—C13	128.9 (2)
F1—C1—C6—C7	−0.2 (4)	N2—C10—C16—C21	−122.1 (2)
C2—C1—C6—C7	179.8 (3)	C9—C10—C16—C21	117.3 (2)
C4—C5—C6—C1	−0.4 (4)	N2—C10—C16—C17	57.9 (3)
C4—C5—C6—C7	−180.0 (3)	C9—C10—C16—C17	−62.8 (3)
N1—O1—C7—C6	−179.5 (2)	C21—C16—C17—C18	−0.4 (4)
C1—C6—C7—O1	179.1 (2)	C10—C16—C17—C18	179.7 (2)
C5—C6—C7—O1	−1.3 (4)	C16—C17—C18—C19	−0.7 (4)
O1—N1—C8—C9	−1.4 (3)	C17—C18—C19—C20	1.1 (4)
O1—N1—C8—C12	177.92 (18)	C18—C19—C20—C21	−0.4 (4)
N1—C8—C9—C14	79.6 (3)	C17—C16—C21—C20	1.0 (4)
C12—C8—C9—C14	−99.7 (3)	C10—C16—C21—C20	−179.0 (2)
N1—C8—C9—C10	−154.8 (2)	C19—C20—C21—C16	−0.6 (4)
C12—C8—C9—C10	25.9 (3)	N2—C11—C22—C23	113.8 (2)
C15—N2—C10—C16	47.3 (3)	C12—C11—C22—C23	−122.5 (2)
C11—N2—C10—C16	167.3 (2)	N2—C11—C22—C27	−66.0 (3)
C15—N2—C10—C9	168.8 (2)	C12—C11—C22—C27	57.7 (3)
C11—N2—C10—C9	−71.1 (2)	C27—C22—C23—C24	0.3 (4)
C8—C9—C10—N2	33.5 (3)	C11—C22—C23—C24	−179.5 (2)
C14—C9—C10—N2	158.9 (2)	C22—C23—C24—C25	0.2 (4)
C8—C9—C10—C16	155.9 (2)	C23—C24—C25—C26	−0.3 (4)
C14—C9—C10—C16	−78.7 (3)	C24—C25—C26—C27	−0.1 (4)
C15—N2—C11—C22	−70.3 (2)	C25—C26—C27—C22	0.7 (4)
C10—N2—C11—C22	167.55 (19)	C23—C22—C27—C26	−0.8 (4)
C15—N2—C11—C12	166.4 (2)	C11—C22—C27—C26	179.0 (2)
C10—N2—C11—C12	44.2 (3)		

### Hydrogen-bond geometry (Å, º)

Cg1–Cg3 are the centroids of the C1–C6, C16–C21 and C22–C27 rings, respectively.

**Table:**

*D*—H···*A*	*D*—H	H···*A*	*D*···*A*	*D*—H···*A*
C7—H7*A*···*Cg*1^i^	0.99	2.96	3.721 (3)	135
C13—H13*A*···*Cg*2^ii^	0.98	2.91	3.577 (3)	127
C18—H18···*Cg*3^iii^	0.95	2.90	3.700 (3)	143
C21—H21···*Cg*2^iv^	0.95	2.51	3.446 (3)	167
C25—H25···*Cg*3^v^	0.95	2.74	3.654 (3)	160

Symmetry codes: (i) *x*+1/2, −*y*+3/2, *z*; (ii) *x*+1, *y*, *z*; (iii) *x*−1/2, −*y*+3/2, *z*; (iv) −*x*, −*y*+2, *z*+1/2; (v) −*x*−1, −*y*+2, *z*−1/2.
